# The Analgesic Effect of Oxytocin in Humans: A Double‐Blind, Placebo‐Controlled Cross‐Over Study Using Laser‐Evoked Potentials

**DOI:** 10.1111/jne.12347

**Published:** 2016-04-25

**Authors:** Y. Paloyelis, C. Krahé, S. Maltezos, S. C. Williams, M. A. Howard, A. Fotopoulou

**Affiliations:** ^1^ Department of NeuroimagingInstitute of Psychiatry Psychology and NeuroscienceKing's College LondonLondonUK; ^2^ Department of Forensic and Neurodevelopmental ScienceInstitute of PsychiatryKing's College LondonLondonUK; ^3^ Research Department of Clinical, Educational and Health PsychologyUniversity College LondonLondonUK

**Keywords:** oxytocin, nociception, analgesia, laser‐evoked potentials, EEG

## Abstract

Oxytocin is a neuropeptide regulating social‐affiliative and reproductive behaviour in mammals. Despite robust preclinical evidence for the antinociceptive effects and mechanisms of action of exogenous oxytocin, human studies have produced mixed results regarding the analgesic role of oxytocin and are yet to show a specific modulation of neural processes involved in pain perception. In the present study, we investigated the analgesic effects of 40 IU of intranasal oxytocin in 13 healthy male volunteers using a double‐blind, placebo‐controlled, cross‐over design and brief radiant heat pulses generated by an infrared laser that selectively activate Aδ‐ and C‐fibre nerve endings in the epidermis, at the same time as recording the ensuing laser‐evoked potentials (LEPs). We predicted that oxytocin would reduce subjective pain ratings and attenuate the amplitude of the N1, N2 and P2 components. We observed that oxytocin attenuated perceived pain intensity and the local peak amplitude of the N1 and N2 (but not of P2) LEPs, and increased the latency of the N2 component. Importantly, for the first time, the present study reports an association between the analgesic effect of oxytocin (reduction in subjective pain ratings) and the oxytocin‐induced modulation of cortical activity after noxious stimulation (attenuation of the N2 LEP). These effects indicate that oxytocin modulates neural processes contributing to pain perception. The present study reports preliminary evidence that is consistent with electrophysiological studies in rodents showing that oxytocin specifically modulates Aδ/C‐fibre nociceptive afferent signalling at the spinal level and provides further specificity to evidence obtained in humans indicating that oxytocin may be modulating pain experience by modulating activity in the cortical areas involved in pain processing.

Oxytocin is a neuropeptide synthesised in the hypothalamus. It is independently released into the bloodstream, exerting hormonal effects on diverse physiological functions 1, and in the central nervous system, exerting neuromodulatory effects on widely distributed oxytocin and vasopressin receptors 2, 3. Oxytocin affects reproductive and social‐affiliative behaviours, including neuroprotective 4 and analgesic 5 roles during birth.

Robust preclinical evidence has demonstrated that exogenous oxytocin has antinociceptive effects and has identified plausible underpinning mechanisms 6. One mechanism involves the engagement of GABA‐mediated inhibitory circuits at the superficial layers of spinal cord dorsal horn neurones, which express oxytocin receptors 7 and receive direct projections from hypothalamic oxytocin neurones 8, 9, reducing Aδ/C‐fibre afferent signalling and ascending nociceptive input 10, 11, 12, 13, 14. Additional mechanisms involve the engagement of the endogenous opioid 15, 16 and cannabinoid 17 systems modulating nociception.

These findings, strengthened by initial evidence from open‐label clinical reports indicating that oxytocin produced analgesia in clinical cases 16, 18, led to the hypothesis that oxytocin has analgesic effects in humans. However, double‐blind, placebo‐controlled studies have produced mixed results. In clinical groups, some reports suggested that a single intranasal oxytocin dose provided headache relief 19 and that an i.v. oxytocin infusion decreased colonic visceral perception in patients with irritable bowel syndrome 20. However, other studies administering intranasal oxytocin daily from 3 to 13 weeks in patients with chronic syndromes did not show any analgesic effects 21, 22.

Experimental studies on healthy volunteers using acute noxious electrical, contact heat and cold‐pressor stimuli have produced further conflicting results. Most reports have not demonstrated any specific antinociceptive properties for oxytocin 23, 24, 25, including a phase 1 open‐label trial using intrathecal administration 26. By contrast, Rash and Campbell 27 reported that intranasal oxytocin reduced the perception of pain intensity and unpleasantness. However, because 70% of the sample correctly identified the treatment that they received (possibly as a result of the use of a saline spray as placebo), the observed analgesic effects of oxytocin could have been partially a result of the potentiating effects of oxytocin on placebo analgesia 24.

In the present study, we investigated the effects of 40 IU of intranasal oxytocin on pain perception and its neural correlates using healthy male volunteers and a double‐blind, placebo‐controlled cross‐over design. We used brief radiant heat pulses generated by an infrared laser that selectively activate Aδ and C‐fibre nerve endings in the epidermis 28. The use of noxious stimuli that specifically engage Aδ/C‐fibre nociceptive afferent signalling in humans may provide a promising tool for illuminating the antinociceptive properties of oxytocin given evidence from electrophysiological studies in rodents suggesting that oxytocin specifically modulates Aδ/C‐fibre nociceptive afferent signalling 10, 29. We predicted that intranasal oxytocin would reduce subjective pain reports and attenuate the amplitude of the N1, N2 and P2 laser‐evoked potentials (LEPs) reflecting the cortical response to nociceptive input 30. Latencies were also examined in an exploratory manner. LEPs reflect temporally distinct cortical processes specifically evoked by the activation of Aδ‐fibres 31 and are differentially modulated by cognitive 32, 33, 34 and pharmacological 35 interventions. Hence, LEPs are ideally suited for investigating the mechanisms underpinning the analgesic effects of oxytocin on pain perception. Given the likely effect of oxytocin on the stress response 36, we also measured salivary cortisol levels to control for stress‐related physiological changes contributing to the predicted analgesic effect.

## Materials and methods

### Participants

Thirteen right‐handed, healthy adult male volunteers participated in the present study (mean ± SD age: 25.69 ± 4.85 years). Participants did not have a history of medical, neurological or psychiatric problems and were screened for current psychiatric conditions using the Symptom Checklist‐90‐R 37 and Beck's Depression Inventory II 38. They did not take any prescribed drugs, tested negative on a urine screening test for drugs of abuse, and consumed < 28 units of alcohol per week and < 5 cigarettes per day. Both parents were white European to reduce genetic background variability 39. Day‐of‐testing lifestyle changes included abstaining from alcohol and heavy exercise for 24 h and not having any beverages or food in the 2 h before the testing session. Data for 18 participants were excluded for showing very low and inconsistent hand temperatures between testing sessions (< 27 °C) as a result of a central heating fault in the laboratory, with six of these not showing any discernible LEPs at baseline recordings (one participant also scored above the cut‐off score of 13 on Beck's Depression Inventory). Although skin temperatures were recorded as a result of their importance for pain perception, the risk of not maintaining consistent skin temperatures within a specific range across active and placebo conditions had not been fully appreciated at the time because, during our pilot studies, the temperature of the laboratory was well controlled. Given the close association between initial skin temperature and the required energy level to reach a given effect, initial skin temperature and consistency across testing conditions in studies investigating the antinociceptive properties of drugs are recognised as important factors that could confound treatment effects 40, 41, 42, 43, 44, 45, 46. We aimed to maintain a dataset of at least N > 11 [comparable to other LEP studies 47, including studies using LEPs to investigate the analgesic properties of drugs 35: N = 12], choosing 27 °C as the cut‐off temperature (a temperature as close to the bottom end of a previously reported typical range as possible, between 28.5 and 33.6 °C) 47. This criterion was set post‐hoc but before the analysis of results. Participants provided their written informed consent and received compensation for their time. King's College London Research Ethics Committee (PNM/10/11‐160) approved the study.

### Nociceptive stimulation

We used an infrared neodymium:yttrium‐aluminum‐perovskite (Nd:YAP; Electronical Engineering, Florence, Italy) laser with a wavelength of 1.34 μm to generate radiant heat pulses. The pulses had duration of 4 ms, were transmitted via an optic fibre cable, and were focused by a lens to a spot diameter of 6 mm at the target site on the dorsum of the left hand. The spot location was changed after each pulse to avoid nociceptor fatigue and sensitisation 48.

### Oxytocin administration

Participants self‐administered 40 IU of intranasal oxytocin (Syntocinon; Novartis, Basel, Switzerland) or placebo (same composition as Syntocinon except for oxytocin). They applied one puff containing 4 IU of oxytocin (or placebo) every 30 s, alternating between nostrils. The administration phase lasted approximately 9 min including a 3‐min rest at the end. Participants reported no side effects during or immediately after the experimental procedure.

### Experimental design

We adopted a double‐blind AB/BA (oxytocin‐placebo/placebo‐oxytocin) cross‐over design with baseline measurements before each treatment 49 to test for treatment effects. We obtained baseline measurements before each treatment because they can contain important background information on each participant and increase the precision of the analyses of treatment effects 49. Participants were randomly allocated to a treatment sequence, receiving each treatment on separate visits (mean ± SD: 13.15 ± 6.05 days apart) at the same time of the day. Of the 13 participants, seven received oxytocin in the first visit and six in the second visit. Infrared laser stimuli were delivered as part of a battery of tasks and questionnaires, and participants were informed that we investigated the effects of a neuropeptide on brain activity and on a range of mental processes. All participants remained blind with respect to the name of the neuropeptide that they received and also the true purpose of the study until debriefing at the end of the second visit.

### Procedures

Participants were seated at a desk facing a computer monitor framed by a screen. The laser equipment and the experimenter were seated behind the screen to prevent visual contact with the participant. Participants rested their left forearm on the desk, extending it through an opening on the screen so that they did not have visual contact with the stimulated hand.

The same testing protocol was followed during each visit. Initially, participants experienced a series of pulses of increasing energy to familiarise them with the equipment, the procedures and the sensations, as well as to determine their individual pain tolerance levels. Participants were asked to focus on the pinprick sensation generated by the activation of the Aδ fibres and rate it on an 11‐point visual analogue scale (VAS) with anchors (0 = 'no pinprick sensation' to 10 = 'the worst pinprick sensation imaginable'). Participants then received three computer‐administered mini‐blocks of pulses, each consisting of all laser intensities, in steps of 0.25 J, up to their individually determined tolerance limit, and were asked to provide a VAS rating after each pulse. These were used to determine separately, for each participant, the experimental stimulus intensity corresponding to a clearly perceived pinprick of moderate intensity, and the control stimulus intensity, corresponding to the energy level at the threshold of perception. The same stimulus intensities were used in the oxytocin and placebo visits for each participant. Subsequently, we fitted the electroencephalogram (EEG) cap. Participants then received two blocks of nociceptive stimuli: one immediately before receiving treatment (‘baseline') and one approximately 45–50 min after treatment onset (‘post‐treatment'), when their EEG was being recorded. Each block consisted of 50 experimental and 20 control trials presented in pseudorandom order. Hand skin temperature was recorded with an infrared thermometer at the beginning of each block. Each trial began with a fixation cross (displayed for 6 s), with a laser pulse being delivered midway (at 3 s). Then the word ‘Rating' appeared on the screen for 3 s as a cue for participants to give their VAS rating orally using the same anchors as above. Each trial ended with a jittered inter‐trial interval (0–5 s; mean trial duration was 11.43 s). The mean ± SD experimental stimulus intensity was 4.12 ± 0.44 J, range 3.35–4.5 J) and the mean ± SD control intensity was 1.83 ± 0.40 J, range 1.5–2.5).

### Salivary cortisol

Salivary samples were obtained via passive drool to measure the unbound form of cortisol 50 at four time points: two samples were obtained during set‐up and averaged to obtain cortisol levels at ‘session onset' (Table 1); one sample at the end of the baseline nociceptive experimental block (before treatment); and a final sample at the end of the post‐treatment experimental nociceptive block (approximately 60–65 min after treatment onset). Salivary samples were frozen at −80 °C until assayed. Free cortisol concentration was measured using the Salimetrics Elisa kit (Salimetrics Europe Ltd, Newmarket, UK), which comprises a competitive immunoassay specifically designed and validated for the quantitative measurement of salivary cortisol. The intra‐assay coefficient of variation (CV) was between 3.35% and 3.65%, and the inter‐assay CV was between 3.75% and 6.41%. The sensitivity of the assay was 0.083 nmol/l.

**Table 1:** Raw Mean Visual Analogue Scale (VAS) Ratings, Mean Local Peak Amplitudes and Latencies of the N1, N2 and P2 Laser‐Evoked Potentials (LEPs), Salivary Cortisol Levels and Skin Temperatures at the Dorsum of the Left Hand from the Baseline and the Post‐Treatment Blocks

	Session onset		Baseline		Post‐treatment	
	Placebo visit, mean ± SD	Oxytocin visit, mean ± SD	Placebo visit, mean ± SD	Oxytocin visit, mean ± SD	Placebo visit, mean ± SD	Oxytocin visit, mean ± SD
N1 local peak amplitude (μV)	–	–	−9.95 ± 4.31	−9.62 ± 3.93	−9.56 ± 4.02	−8.16 ± 2.88
N1 local peak latency (ms)	–	–	186.14 ± 21.64	192.37 ± 21.18	185.34 ± 18.52	193.80 ± 19.41
N2 local peak amplitude (μV)	–	–	−12.58 ± 7.10	−12.01 ± 6.76	−11.65 ± 7.39	−9.67 ± 5.45
N2 local peak latency (ms)	–	–	217.79 ± 19.68	218.72 ± 25.53	215.30 ± 21.08	225.41 ± 23.65
P2 local peak amplitude (μV)	–	–	11.17 ± 5.56	11.29 ± 4.75	10.68 ± 5.22	9.68 ± 3.10
P2 local peak latency (ms)	–	–	333.28 ± 28.31	335.91 ± 36.69	334.70 ± 27.37	339.72 ± 34.36
VAS ratings	–	–	3.83 ± 1.47	3.32 ± 1.29	3.35 ± 1.44	3.12 ± 1.50
Salivary cortisol (nmol/l)	5.56 ± 3.34	5.75 ± 2.78	4.75 ± 3.07	4.14 ± 2.18	3.74 ± 2.44	4.16 ± 2.73
Skin temperature (°C)	–	–	31.09 ± 1.99	30.69 ± 1.80	30.58 ± 1.82	29.79 ± 2.10

### LEPs

Brief radiant heat pulses generated by an infrared laser selectively activate Aδ‐ and C‐fibre skin nociceptors and generate a series of transient, time‐locked brain responses 51 that appear as deflections in the EEG and specifically reflect the activation of Aδ fibres 52. LEPs comprise a negative‐positive deflection, maximal at the scalp vertex (N2‐P2 wave, peaking at 200–350 ms when stimulating the hand dorsum), and a preceding smaller negative deflection maximal at the contralateral temporal electrodes (N1 wave, peaking at approximately 160 ms) 48.

### EEG recording

EEG data were collected using a 64‐channel Neuroscan Quik‐cap elasticised cap with passive AgCl electrodes and recorded using scan, version 4.3 (Compumedics, Ltd, Charlotte, NC, USA). Data were collected from 64 electrodes positioned on the scalp in accordance with the International 10–20 system. A bipolar electrode on the earlobes was used as the recording reference and additional electrodes on the mastoids were used to serve as reference electrodes for the analyses. The electrooculogram was recorded by placing a bipolar electrode above and below the right eye and two electrodes to the left and right of each eye. Impedances were kept below 5 kΩ. The data were sampled at 5000 Hz.

### EEG analysis

EEG data were processed using the matlab (R2011a; MathWorks Inc., Natick, MA, USA) open‐source toolboxes eeglab 53 and erplab 54. We focused our analyses on the experimental trials because the control trials were included to decrease the predictability of the experimental stimuli. EEG data were down‐sampled to 250 Hz and re‐referenced using the averaged recordings from the mastoid electrodes before applying a high‐pass filter of 0.4 Hz. The EEG was then segmented into −500‐ to 1000‐ms epochs relative to the onset of the stimulus and a 30 Hz low‐pass filter was applied. Epochs from the baseline and post‐treatment blocks were concatenated and trials with gross artefacts (exceeding 400 μV) were removed. Ocular artefacts were removed using independent component analyses (‘runica' algorithm) in eeglab. Prototype blinks and saccades were identified following visual inspection and the corrmap utility 55 was used to identify matching artefacts across the datasets for removal. Finally, epochs containing artefacts exceeding ± 100 μV were removed, then epochs were resegmented to −200 to 800 ms and baseline‐corrected (−200 to 0 ms). Fewer than 10% of trials were removed from each dataset. The erplab measurement tool was used to measure the local peak amplitude and latency of the LEPs. Average waveforms for each component time‐locked to stimulus onset were computed for each block (baseline, post‐treatment) and treatment condition (oxytocin, placebo). The peak‐to‐baseline amplitude and the latency of the N2 (latency window: 100–350 ms) and P2 (latency window: 250–420 ms) components were measured at the Cz electrode using an average reference, and those of the N1 component (latency window: 0–270 ms) were measured at the C4 electrode (contralateral to the stimulated hand), using the Fz electrode as reference.

### Statistical analysis

Our primary outcome variables were the mean VAS ratings and the mean local peak amplitudes of the N1, N2 and P2 LEPs for each block. Component latencies and salivary cortisol levels (nmol/l) were secondary outcome variables. We tested for treatment effects (oxytocin versus placebo) using the analysis of covariance (ancova) approach described by Senn 49 and Metcalfe 56 for the analysis of AB/BA cross‐over designs with baseline measurements before each treatment. If T = active treatment (intranasal oxytocin), C = control treatment (placebo), X_T_ and X_C_ are the corresponding baseline measurements, and Y_T_ and Y_C_ the corresponding post‐treatment measurements, using the regression command in stata, version 13 (StataCorp, College Station, TX, USA), we implemented the ancova model: (Y_Ti _− Y_Ci_) = β_T_ + γ(X_Ti _− X_Ci_) as described by Senn 49 and Metcalfe 56, separately, for each outcome variable. The intercept term in this model tests for the treatment effect. In the regression model, we also included a binary explanatory variable representing treatment sequence (AB or BA) for each measure where there was a significant period effect (which might reflect a general tendency, irrespective of treatment). We tested for period effects (i.e. changes in measurements across visits, obtained by subtracting post‐treatment measurements at the first visit from post‐treatment measurements at the second visit) by regressing period differences on treatment sequence. Additionally, we computed baseline‐corrected basic estimators of treatment effects by subtracting the corresponding baseline differences weighted by their regression slope. As a control, we tested for treatment effects on skin temperatures using the same approach; we also used paired‐sample t‐tests to check separately for skin temperature differences between baseline and post‐treatment measurements under oxytocin and placebo. Finally, we tested for time effects on salivary cortisol levels (to investigate whether the administration of the nociceptive stimuli increased cortisol levels) using a 3 (Time: session onset, before treatment, post‐treatment) by 2 (Treatment: oxytocin, placebo) analysis of variance (anova) model implemented in stata, version 13 (StataCorp) using the ‘regression' command and robust variance estimation (‘cluster' option) to correct for data dependence 5 (as a result of the within‐subjects factors). We conducted statistical inferences using nonparametric bootstrapping estimation (1000 repetitions), which does not make distributional assumptions on the data 57.

## Results

Table 1 presents raw mean VAS ratings, mean local peak amplitudes and latencies of the N1, N2 and P2 LEPs, salivary cortisol levels and skin temperatures at the dorsum of the left hand from the baseline and the post‐treatment blocks. Table 2 summarises the results of the regression analyses examining the effects of intranasal oxytocin, compared to placebo, for each outcome variable.

**Table 2:** Regression Analyses Showing the Effect of Intranasal Oxytocin (Compared to Placebo) for Each Outcome Variable

	b^a^	95% CI^b^	SE	Z	P	Estimated treatment effect^c^	d^d^
N1 local peak amplitude	1.65	0.11, 3.20	0.79	2.09	0.036	1.77	0.63
N1 local peak latency	1.06	−5.49, 7.62	3.34	0.32	0.75	3.08	0.26
N2 local peak amplitude	1.79	0.27, 3.32	0.78	2.30	0.021	1.98	0.73
N2 local peak latency	10.03	3.25, 16.81	3.46	2.90	0.004	10.11	0.83
P2 local peak amplitude	−1.07	−2.51, 0.36	0.74	−1.46	0.145	−0.99	−0.38
P2 local peak latency^e^	1.99	−3.70, 7.68	2.90	0.69	0.493	5.02	0.49
VAS ratings^e^	−1.27	−2.25, −0.29	0.50	−2.53	0.011	−0.23	−0.20
Salivary cortisol	0.62	−0.74, 1.98	0.69	0.90	0.37	0.42	0.18
Including initial session recordings	0.52	−0.71, 1.76	0.63	0.83	0.41	0.42	0.20
Skin temperature	−0.51	−1.40, 0.38	0.45	−1.13	0.26	−0.78	−0.46

VAS, visual analogue scale.

a The presented statistics correspond to the intercept of the regression line that estimates the effect of interest.

b 95% confidence interval for the b coefficient.

c Estimated marginal mean for the treatment effect.

d Cohen's d (estimated treatment effect/SD) 90.

e Regression models included treatment sequence (AB/BA) as a covariate because of the presence of significant period effects, as described in the Statistical analysis and reported in the Results.

### LEPs and VAS scores

**Figure 1:**
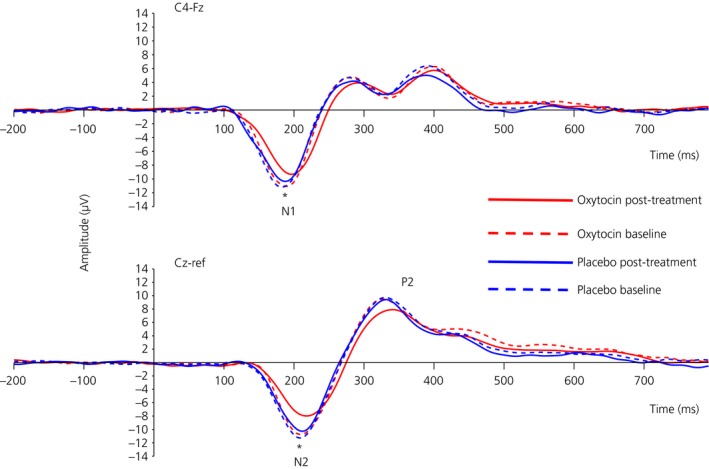
Grand mean of laser evoked potentials (LEPs) after stimulation of the left‐hand dorsum (N = 13) at baseline and after receiving treatment (oxytocin or placebo). x‐axis: time (ms); y‐axis, amplitude (μV). Top: N1 wave recorded at the temporal region contralateral to the stimulated site (C4 versus Fz). Bottom: N2/P2 wave recorded at the vertex (Cz versus average reference). Full waveforms are LEPs obtained after treatment (oxytocin: red; placebo: blue). Dashed waveforms are LEPs obtained before treatment (baseline). There was a significant reduction in N1 local peak amplitude (P = 0.036) following oxytocin treatment (compared to placebo and controlling for baseline). There was also a significant reduction in N2 local peak amplitude (P = 0.021) and an increase in N2 local peak latency (P = 0.004) following oxytocin treatment (compared to placebo and controlling for baseline). There was no significant effect of oxytocin treatment (compared to placebo) on the P2 wave. *P < 0.05.

Intranasal oxytocin significantly reduced subjective VAS ratings, as well as N1 and N2 local peak amplitudes, and increased N2 local peak latency (Fig. 1 and Tables 1 and 2). Baseline‐corrected treatment effect estimators for VAS ratings were significantly correlated with the N2 (r = −0.57, P = 0.042) but not the N1 (r = −0.47, P = 0.11) local peak amplitudes, although both correlations were moderate and in the expected direction (i.e. the greater the oxytocin‐induced reduction in VAS ratings the greater the oxytocin‐induced attenuation of the LEP amplitude). Period effects were significant only for the post‐treatment P2 local peak latency (b = 32.62, SE = 11.82, Z = 2.76, P = 0.019) and the VAS ratings (b = −1.55, SE = 0.65, Z = 2.40, P = 0.035) and hence included in the corresponding regression models (as explained in the Statistical analysis).

### Skin temperature

Skin temperatures ranged within expected levels for studies using laser‐generated radiant heat stimuli 47. There was no treatment effect on skin temperatures (Table 2). Furthermore, skin temperatures did not differ between treatments across the baseline (t_12_ = 0.51, P = 0.62) or the post‐treatment blocks (t_12_ = 1.10, P = 0.29).

### Salivary cortisol levels

There was no treatment effect on salivary cortisol levels (Table 2). An anova showed a significant effect of Time $\chi_{2}^{2}$ = 11.14, P = 0.004, with salivary cortisol levels decreasing between session onset and post‐treatment block (Table 1). Post‐hoc tests showed that salivary cortisol levels were significantly lower at the end of the post‐treatment block compared to session onset ($\chi_{1}^{2}$ = 11.12, P < 0.001) but not compared to the end of the pre‐treatment block ($\chi_{1}^{2}$ = 3.02, P = 0.082); no significant difference was observed between the end of the pre‐treatment block and session onset ($\chi_{1}^{2}$ = 2.01, P = 0.16).

## Discussion

In this double‐blind, placebo‐controlled, cross‐over study, we stimulated Aδ‐ and C‐fibre skin nociceptors in the dorsum of the hand using infrared laser radiant heat stimuli in healthy male volunteers. We found that a single dose of intranasal oxytocin (40 IU) attenuated both perceived pain intensity and laser‐evoked cortical responses. Specifically, intranasal oxytocin attenuated the local peak amplitude of the N1 and N2 (but not of P2) LEPs, and increased the latency of the N2 component. Importantly, for the first time, the present study reports an association between the analgesic effect of oxytocin (reduction in subjective pain ratings) and the oxytocin‐induced modulation of cortical activity following noxious stimulation (attenuation of the N2 LEP). These effects indicate that oxytocin modulates neural processes contributing to pain perception and are discussed in turn below.

### Subjective ratings

We observed a 7% reduction in subjective pain intensity ratings following intranasal oxytocin (compared to placebo). This effect is smaller but comparable to a recent study showing that 40 IU of intranasal oxytocin induced an 11% reduction in intensity ratings following the application of cold‐pressor pain on healthy volunteers 27. Our studies differ not only in terms of pain modality, but also in the timing of the intervention following oxytocin treatment. This question has only recently began to be addressed, with a recent study suggesting changes in regional cerebral blood flow (rCBF) in the resting state peak at 30–42 min from the end of intranasal oxytocin (40 IU) administration 58. Indeed, in the study reporting the larger reduction in pain intensity ratings 27, the intervention commenced at 20 min post‐oxytocin treatment and partially overlapped with the temporal window during which the effects of intranasal oxytocin in the brain are maximal, whereas, in the present study, the intervention spanned 45–60 min post treatment. Thus, our study confirms that future research should consider the temporal dynamics of the pharmacodynamic effects of oxytocin in order to illuminate its antinociceptive properties 58.

### LEPs and mechanisms mediating the effects of oxytocin on pain

We further observed that intranasal oxytocin specifically modulated the N1 and N2 but not the P2 LEPs. Although the functional significance of LEPs is not yet clearly understood 59, the cortical processes generating LEPs are largely related to the perception of salient changes in the sensory environment 59, 60, and contribute to the perception of pain 61, 62. The early N1 component, reflecting the activity of cortical generators in the contralateral operculoinsular and primary somatosensory cortices 30, 63, is largely driven by the magnitude of the ascending nociceptive input, representing an early stage of sensory processing before the perceptual outcome of the nociceptive input is determined 48, 64, 65. The later N2 and P2 components reflect cortical generators in the insular and anterior cingulate cortices 30, 63, and also relate to the intensity of noxious stimuli, although they largely represent cortical mechanisms that determine the subjective experience of pain 48.

Our observation that intranasal oxytocin reduced the amplitude of the N1 and N2 but not the P2 cortical responses suggests that it affects specific processes contributing to pain experience. First, the reduction in N1 and N2 amplitudes (and increase of N2 latencies) is consistent with effects of oxytocin at spinal levels, affecting the coding of noxious stimulus intensity and the ensuing experience of pain. Indeed, animal studies have shown that oxytocin can specifically reduce Aδ/C‐fibre afferent signalling and ascending nociceptive input at the level of spinal cord dorsal horn neurones by engaging GABA‐mediated inhibitory cellular mechanisms 10, 11, 12, 13, 14, 29. Additionally, oxytocin may be modulating the pain experience in humans by modulating activity in cortical areas involved in pain processing. In humans, intranasal oxytocin has been shown to increase rCBF, and hence neuronal activity, in cortical areas directly implicated in the processing of nociceptive input and the experience of pain, as well as in the descending modulation of nociceptive input, such as the frontoparietal opercula, the insula and the anterior cingulate cortex 58, 66, 67. Changes in neuronal activation in these cortical areas have been shown to modulate the evoked brain responses to transient nociceptive input 68. For example, the observation of emotionally conflicting information that activates the anterior cingulate cortex modulates the perception of pain and specifically prevents the full expression of the N2 component (reducing its amplitude) 68. Consistent with this and the role of N2 in the experienced pain intensity, we observed that the oxytocin‐induced reduction in pain ratings correlated with the oxytocin‐induced reduction in N2 amplitude. The lack of intranasal effects of oxytocin on the later P2 component suggests that it may not have influenced the perceived salience of the noxious stimuli. The P2 component has been shown to be modulated specifically by factors reflecting the salience of noxious stimuli (e.g. stimulus probability) 33, 59, 69 and to reflect multimodal (rather than somatosensory‐specific) processes 61.

The intranasal dose administered in the present study has been associated with changes in central function in humans 58, and similar or smaller doses of oxytocin or vasopressin (24–48 IU for oxytocin; 40–80 IU for vasopressin) have been associated with elevations in peptide levels in the cerebrospinnal fluid in humans or macaques of no more than approximately 55 pg/ml in actual levels 70, 71, 72, 73, 74, or of up to 0.005% of the administered dose 75. It is possible that the observed effects in the present study could be at least partially explained by elevations in central levels of oxytocin, although it is not clear to what extent this elevation reflects endogenous or exogenous oxytocin and, if the latter, what the mechanisms of absorption may be. However, it remains unknown how much oxytocin must enter the brain in primates for a behavioural effect to be observed; in smaller animals, 1 ng has been reported as the lowest i.c.v. dose shown to elicit a behavioural effect 75. Additionally, it is also possible that the antinociceptive effects of intranasal oxytocin observed in the present study are explained by the activation of peripheral receptors, mainly the vasopressin 1A receptor 76. Future studies will need to specifically address the mechanism mediating the antinociceptive effects of oxytocin in humans; for example, by administering antagonists to block peripheral oxytocin or vasopressin receptors 75.

### Impact on the stress response

A further mechanism postulated to potentially mediate intranasal effects of oxytocin on pain sensitivity in humans involves the mitigation of psychological factors such as negative mood, anxiety and the stress response to pain 27. In the present study, we did not observe an effect of oxytocin on salivary cortisol levels. This might be because our participants did not find our pain paradigm (involving a highly controlled stimulus) sufficiently stressful in that there was a reduction in salivary cortisol levels over time irrespective of treatment condition. However, a review of human studies examining the effect of oxytocin on cortisol suggests that oxytocin may exert both anxiogenic and anxiolytic effects, depending on a variety of individual and context variables 77, 78, which are not always reflected in changes in cortisol levels 79, 80. Furthermore, existing studies have either failed to show a mitigating effect of oxytocin on negative mood 27 or have shown that such an effect was not related to the pain experience 23.

### Mediating social effects on pain

The analgesic effects of exogenous oxytocin in humans identify the oxytocin system as a plausible neural mechanism for the transduction of the effects of social support into the neural and physiological changes that modulate the experience of pain in humans 81, and such a hypothesis needs to be addressed in future research. Social factors, such as the supportive presence of others, have been shown to modulate the experience of pain 82. Interaction with other humans, including warm interpersonal contact 83, 84 or the perception of trust 85, can result in the release of endogenous oxytocin. Preclinical studies demonstrate that manipulations involving bodily contact such as massage can induce anti‐nociceptive effects in rodents similar to those elicited by exogenous oxytocin, by triggering the endogenous oxytocin system 86.

### Limitations

A number of limitations characterise the present study. First, we focused on male participants because some degree of sexual dimorphism in the oxytocin system may be expected 87. Hence, our findings cannot be readily extrapolated to women. Second, we used the maximal oxytocin dose safely administered to humans 88 and a high intensity of the noxious stimulus because our aim was to assess the presence of analgesic properties of intranasal oxytocin in humans. Future studies should systematically examine a wider range of dosages and intensities of noxious stimuli, as well as include clinical populations, aiming to characterise the analgesic properties and potential clinical relevance of intranasal oxytocin. Third, future studies should explicitly investigate whether oxytocin influences the salience of noxious stimuli and whether it modulates pain‐specific processing or sensory processing in general 23. Fourth, because we did not measure heart rate variability in the present study, we could not determine whether the analgesic effects of intranasal oxytocin were at least partially mediated by oxytocin‐induced modulation of heart rate reactivity to pain 27. Last but not least, although the present study investigated an effect of oxytocin on a phenotype that could be reasonably expected on the basis of animal research, given the small sample size, it is possible that the observed treatment effect is overestimated 89. Our findings will need to be replicated in future studies that are adequately powered to detect even smaller treatment effects.

## Conclusions

By selectively stimulating subcutaneous Aδ‐ and C‐fibres with an infrared laser and recording the EEG in participants, we provide preliminary evidence indicating that a single intranasal oxytocin dosage (40 IU) attenuated pain intensity ratings and differentially modulated the ensuing cortical LEPs in humans. Our findings are consistent with robust preclinical evidence on the antinociceptive properties of oxytocin and highlight potential neural mechanisms mediating the analgesic effects of oxytocin in humans.
